# What makes us act together? On the cognitive models supporting humans’ decisions for joint action

**DOI:** 10.3389/fnint.2022.900527

**Published:** 2022-08-03

**Authors:** Arianna Curioni

**Affiliations:** Department of Cognitive Science, Central European University, Vienna, Austria

**Keywords:** joint action, cooperation, decision, utility, coordination, reward

## Abstract

We face tasks every day that we can solve alone but decide to solve together with others. When do we choose to act together vs. alone? How long do we persist in working together when doing so is difficult? Do we prefer to act together when times are uncertain? An open question in joint action research is under what conditions humans prefer to act together or alone to achieve a certain goal, and whether their preference is based on a utility calculus that takes into account the costs and benefits associated with individual and joint action alternatives. Research on cooperation reveals that frequent engagement in joint activities provides high survival benefits, as it allows individuals to achieve goals together that are otherwise unavailable. Yet, survival advantage does not wholly explain the reasons for human cooperative behavior. In fact, humans are motivated to cooperate even when it is not necessary to achieve an outcome. Research in cognitive science suggests that navigating the potential costs of joint actions is a challenge for humans, and that joint actions might provide individuals with rewards that go beyond the achievement of instrumental goals. We here address the influence of key factors on the decision to engage in joint action, such as the coordination costs arising when acting together compared to alone and the social and instrumental rewards expected when acting together compared to alone. Addressing these questions will provide critical insight for the design of cognitive models of human decisions for cooperation.

## Introduction

In this article, we address the question of how humans decide whether to act alone or jointly to achieve instrumental goals. To do so, we first discuss whether humans have, unlike other animals, a preference for cooperative goal achievement by discussing theory and findings from cognitive, developmental, and comparative psychology. We then discuss existing models of human decision-making in individual action scenarios and analyze whether and how such models can be applied to the case of deciding between individual and joint goal achievement. Here, we focus on utility-based models and unpack what may be the factors that influence the computation of utility of individual and joint actions, namely the costs and benefits individuals expect to be associated with these two action alternatives. Finally, we outline how the proposed approach can be used to address several outstanding questions about human cooperative decision-making.

## Do humans prefer to act together rather than alone?

Imagine you moved to a new house, and it is now time to carry all your books up the stairs. You packed them all in 10 heavy boxes. You could carry all the boxes by yourself, or you could ask a friend to help you carry them up. If you choose to do it with your friend, you will need to coordinate your steps and your arms while navigating the narrow staircase while sharing the weight of each box. If you choose to do it by yourself, you will carry more weight but won’t have to deal with any coordination problem. Regardless of your choice, the outcome will be the same: all your books will be in your flat. How will you decide whether to do it alone or together with your friend?

To find an answer to this question, we will here inquire into the factors that influence humans’ decisions to act alone or jointly with a partner to achieve the desired outcome.

Extensive research on human cooperation shows that we are the most motivated cooperative agents among many other species. In fact, only humans engage in large-scale cooperation with non-kin individuals and strangers (Herrmann et al., 2007; Boyd and Richerson, 2009; Tomasello et al., 2012). Evidence from comparative and developmental science indicates that humans start to spontaneously engage in cooperative activities from early on in development (Warneken and Tomasello, 2007; Warneken et al., 2012). Around the 3rd year of life, they also prefer to achieve their goals by acting together with a partner rather than alone (Rekers et al., 2011). Although chimpanzees, our closest evolutionary relatives, can perform cooperative activities, they do so only when the desired outcome cannot be achieved by a single individual or when cooperation offers greater rewards (Hrdy, 2009; Jaeggi et al., 2010; Bullinger et al., 2011; Rekers et al., 2011). Other prosocial acts such as food transfer, teaching, and cooperative child care—foundational of humans’ social life—are more commonly observed in cooperative breeders like new world monkeys, with some exceptions of altruistic behavior in bonobos (Brotherton et al., 2001; Burkart and van Schaik, 2010; Tan and Hare, 2013). However, these kinds of behaviors are very rare in this species, and they are usually carried out by adult individuals only.

Much work has investigated the biological and cultural factors explaining the selection of altruism and cooperation in humans (Nowak, 2006; Warneken and Tomasello, 2009; Melis and Semmann, 2010). One prominent hypothesis is that cooperation evolved to facilitate foraging, hunting, and breeding. Specifically, social interdependence in these behaviors allowed humans to achieve goals that were not available to individuals alone, thus providing them with an essential survival benefit (Tomasello and Gonzalez-Cabrera, 2017).

Nevertheless, in some instances, although cooperation supports the welfare of the group, it may be very costly for the individuals involved. This produces a social dilemma: how much cost are individuals willing to pay to benefit other members of their group? Decision strategies in these scenarios have been investigated at length using various kinds of experimental paradigms modeling social dilemmas, such as the Prisoner’s Dilemma and the Public Goods Game (Cooper et al., 1996; Sanfey, 2007; Camerer, 2011; Rand et al., 2014) or the Stag Hunt game (Skyrms, 2004; Duguid et al., 2014). The latter is interesting because it models a scenario where cooperation benefits all individuals involved. Two or more individuals hunt for hares—prey that can be captured alone—and then spy on a stag, which represents a more valuable food resource than a hare but can only be captured by individuals together. Since it is in everyone’s interest to hunt the stag as it is more nutritious, individuals choose to collaborate to capture the stag and fairly share the spoils. This represents a prototypical situation where cooperating with a partner will ensure mutual benefits; therefore, we can expect humans to show a stable preference for cooperation over individual goal achievement in such scenarios.

Yet human cooperative behavior cannot be explained by mutual advantage only. In fact, many of the joint activities that we engage in do not provide an easily measurable instrumental payoff, neither to the individual nor to the group. We travel with friends although making decisions together is challenging; we promote and value teamwork even if it does not always result in more efficient outcomes. We design art and entertainment such as collective music making or team sports, based on collaborative acts that are often very complex and costly. To put it simply, many everyday decisions to engage in joint activities cannot be explained by the instrumental payoff.

## A utility model of acting together

There are several open questions about the decision-making processes behind cooperative choices that do not provide instrumental benefit: Why and how do we decide to act together when it is *not necessary nor instrumentally advantageous*? Here, we will discuss potential models for the decision for cooperative joint actions, i.e., goal-directed instrumental actions that are carried out by two or more agents who coordinate in space and time to achieve individual and shared goals (Sebanz et al., 2006).

To decide among action alternatives, we apply intuitive cost-benefit analyses where we weigh the potential rewards of each option against anticipated costs (Kahneman and Tversky, 2013). When making such comparisons, we intuitively use the common currency of costs and rewards, for example, when deciding whether to take the car or the bicycle to go to work or which school to send our children to Ruff and Fehr (2014). These computations can be influenced by many factors: our energetic levels, preferences, the potential risks associated with each choice, the time delay to the outcome or moral considerations. But all things considered, we can expect human adults to minimize their effort and maximize their welfare in everyday decisions, although their reasoning may often depart from perfect optimization (Von Neumann and Morgenstern, 2007; Tversky and Kahneman, 1974; Gilovich et al., 2002). From the perspective of action planning and motor control, the performance of goal-directed actions is also guided by principles of optimization and utility (Wolpert and Landy, 2012; Cisek and Pastor-Bernier, 2014). In fact, the selection of action plans is determined by prior knowledge of actions’ costs and outcomes (Todorov and Jordan, 2002).

Interestingly, principles of optimality in the action domain are also pivotal to infants’ ability to attribute goals to the actions of others: the expectation that other agents will perform actions in the most efficient way constitutes one of the pillars of humans’ ability to understand intentional actions and later develop optimal action planning (Gergely and Csibra, 2003; Spelke and Kinzler, 2007; Southgate and Csibra, 2009). In the developmental psychology, utility models have been recently adopted to explain how infants and children understand, compare, and select individual actions in social contexts (Naive Utility Calculus model, Jara-Ettinger et al., 2016, 2020; Liu et al., 2019; Bridgers et al., 2020; Lucca et al., 2020). Although little is known about whether and how adults represent the utility of joint actions, recent work demonstrated that when they are involved in coordinated joint actions with a partner, adults consistently prioritize joint efficiency over individual efficiency, i.e., individuals prefer task solutions that minimize the action costs for both co-actors (Török et al., 2019, 2021). While this suggests that individuals are capable of computing and comparing individual and joint utility, it is not clear whether individuals expect the same kinds of costs and rewards to be associated with the two action alternatives. In fact, Curioni et al. (2022) show that when individuals are faced with the decision of whether to achieve a goal individually or jointly, they show a strong preference for joint actions, although this action alternative is the least efficient in terms of time and success rate. These findings support the idea that other factors, beyond instrumental payoff, may play an important role when comparing individual and cooperative actions. Therefore, it seems useful to include more than just instrumental factors into a utility model of joint actions, to predict individuals’ decisions in these scenarios. In the following sections, we will consider the specific costs and rewards individuals may incur when acting together as key factors of influence on human decisions, and outline how they could be integrated within individuals’ utility model of joint action.

### The costs of acting together

Here, we focus on the decision-making that occurs when people choose between acting alone or together with a partner to achieve the desired outcome. One crucial step is to identify the costs and rewards individuals may expect from the two action alternatives—alone or together (Bridgers et al., 2020).

The planning and performance of individual and joint actions imply the recruitment of distinct cognitive and behavioral processes. When engaging in coordinated actions with a partner, individuals incur various kinds of coordination costs. In fact, a seemingly effortless and smooth joint action always hides a complex coordination problem. To perform joint actions, individuals need to coordinate their actions and decisions in space and time (Sebanz et al., 2006; Vesper et al., 2010) and engage in costly cognitive computations, such as mentalizing or representing conflicting perspectives (Moll and Kadipasaoglu, 2013; Freundlieb et al., 2016). They also recruit dedicated prediction and monitoring processes (Kourtis et al., 2013; Loehr et al., 2013; Moreau et al., 2022; Sacheli et al., 2022) ensuring coordination at the millisecond level. Furthermore, without dedicated representations of the task, goal, and actions (Newman-Norlund et al., 2007; Sacheli et al., 2018), coordinating actions toward a common goal would be impossible. When solving a task together, co-actors engage in turn taking and task distribution, incurring significant costs in terms of cognitive control, inhibition, task switching, and loss of agency (Koch et al., 2010; Loehr, 2022). Evidence also suggests that performing joint actions is cognitively taxing, as task distribution among cooperation partners significantly increases cognitive load (Wahn and Kingstone, 2020; Wahn et al., 2021).

The costs of coordination can also be observed at the level of action performance. Evidence from research on joint music making indicates that spatial and temporal synchronization requires fine sensorimotor skills and predictive abilities (Keller et al., 2014). Furthermore, when engaged in joint actions, individuals modify their actions online to communicate their goals and intentions. These modifications are costly deviations from optimal action performance that support coordination, action understanding, and goal disambiguation (Vesper and Richardson, 2014; Candidi et al., 2015; McEllin et al., 2018; Curioni et al., 2019; Pezzulo et al., 2019). Beyond cognitive and behavioral demands, coordination can be costly also at the level of payoff. In fact, engaging in cooperation can heighten the uncertainty of action outcomes, requires more deliberation, and expose individuals to potential defection, freeriding, and punishment from cooperation partners (Fehr and Gächter, 2002; Rand and Nowak, 2013; Bear and Rand, 2016; Fehr and Schurtenberger, 2018).

The exact nature and magnitude of coordination costs depend on the specific joint action performed and the kind of coordination required. For instance, when carrying a box together, an agent will incur temporal and spatial coordination costs that are not present when carrying a box alone. These costs may be particularly high if, for example, the partner is very clumsy or much taller than the agent, or the box is shaped so that it is difficult to grasp from the sides. This suggests that the specific action costs of the task at hand are also a crucial factor of influence when assessing the costs of acting together.

### The benefits of acting together

The way costs of the individual or joint actions can be evaluated is influenced by the expected or experienced rewards that can be achieved through such actions. In cases when cooperating with a partner allows the achievement of goals that are not available to individuals alone, joint actions provide benefits that outweigh their cost. However, when a joint action is not necessary to achieve an outcome, the benefits associated with outcome achievement *per se* cannot explain why individuals would prefer it. This begs the questions of whether humans attribute a specific reward value to joint actions that go beyond instrumental benefits. If so, can we capture this value in a model of joint action utility?

Evidence from cognitive neuroscience research shows that decisions in the social domain are driven by reward-related computations, similar to non-social decisions (FeldmanHall and Shenhav, 2019). Furthermore, being involved in social interaction, attending to social stimuli, and being addressed by others activate reward-related brain processes that influence motivational control and decisions in social interactions (Fehr and Camerer, 2007; Ruff and Fehr, 2014). This implies that because social stimuli may be particularly rewarding for us, we will prefer them over other kinds of stimuli, and we may be more motivated to invest the effort to obtain them. Theories on cooperation show that individuals derive various benefits from engaging in cooperation—from affiliation to reputation and social status, to future reciprocation (Fehr and Fischbacher, 2004). Humans also assign a positive moral value to cooperative actions such as helping, committing to others, and investing effort in the interaction (Szeìkely and Michael, 2018; McEllin and Michael, 2022). Importantly, a possible benefit that individuals may expect from acting cooperatively is sharing responsibility, i.e., minimizing the individuals’ psychological and material burden, both when performing collective decisions and when facing the negative outcomes of those decisions (El Zein et al., 2019).

From a cognitive perspective, if individuals were more motivated to engage in joint action, this could have a boosting effect on their cognitive performance (Botvinick and Braver, 2015; Yee and Braver, 2018; Westbrook et al., 2020). Relatedly, another important factor that can influence the decision to act together is the motivational drive for the social relationship with the interactive partner: in fact, evidence indicates that interpersonal perception modulates interactive behavior (Sacheli et al., 2012) and that the identity of the joint action partner can influence both decisions and performance at a cooperative task (Boukarras et al., 2021). Moreover, studies on macaques suggest that interacting with closely related individuals reduces cortisol levels (Stocker et al., 2020). These suggest that both the identity and the perception of the interactive partner can strongly influence the decision to act together.

Altogether, findings and models from research in cognitive neuroscience indicate that individuals attribute a reward value to social stimuli and to social interactions. Do individuals also attribute a reward value to the engagement in a joint action? If so, can this reward value be integrated into a utility model of acting together? We propose that a utility model of acting together that accounts for such reward would allow us to test outstanding questions and improve our understanding of humans’ decisions in cooperation scenarios.

## How do we decide to act together? Implications for future research

As discussed above, the cognitive processes that support human cooperative preferences beyond instrumental utility remain to be explored. Here, we argue that a useful approach to address these questions is to develop a utility model of acting together that can account for the specific coordination costs and rewards that individuals expect to incur when performing joint actions. We would like to explore fundamental questions for future research.

How are coordination costs and rewards integrated in a utility model of acting together, and how do they influence humans’ decisions in cooperation scenarios? The likelihood of task failure, the cost of error monitoring, and the perceived cognitive load during the task may be crucial factors in the decision for joint action, but may have different weights in the utility calculus.

1. Does interaction history with a partner influence the reward value assigned to joint actions?
2. Is there a common currency for the utility of instrumental and joint actions in social contexts? One possibility is that individual costs and rewards of actions are integrated into the utility calculation of joint actions, or instead is the action context that determines the rules governing utility calculations. For example, once a particular action context is identified (“joint action”), a dedicated utility scale applies. Our framework provides the means to test these alternative hypotheses and advance our understanding of the utility model of joint actions.
3. Interpreting others’ actions and inferring preferences from their choices are indispensable tools for navigating social environments successfully. What heuristics for human behavior should be implemented in artificial agents to maximize success in human–machine interaction? Since human agents could be biased toward acting together against instrumental utility or could be biased toward acting together because it is more rewarding, this suggests that pure optimization may not be a successful heuristic to predict decisions performed by agents in interactive contexts. This highlights the need of a computational model of human decisions for joint action that accounts for heuristics, preferences, and biases better characterizing our social behavior.

## Data availability statement

The original contributions presented in this study are included in the article/supplementary material, further inquiries can be directed to the corresponding author/s.

## Author contributions

The author confirms being the sole contributor of this work and has approved it for publication.
